# Targeting triple-negative breast cancer: A clinical perspective

**DOI:** 10.32604/or.2023.028525

**Published:** 2023-05-24

**Authors:** LAZAR S. POPOVIC, GORANA MATOVINA-BRKO, MAJA POPOVIC, KEVIN PUNIE, ANA CVETANOVIC, MATTEO LAMBERTINI

**Affiliations:** 1 Department of Medical Oncology, Oncology Institute of Vojvodina, Sremska Kamenica, Serbia; 2 Faculty of Medicine, University of Novi Sad, Novi Sad, Serbia; 3 Department of General Medical Oncology and Multidisciplinary Breast Centre, Leuven Cancer Institute, University Hospitals Leuven, Leuven, Belgium; 4 Department for Oncology, Medical Faculty Nis, University of Nis, Nis, Serbia; 5 Clinic of Oncology, Clinical Centre Nis, Nis, Serbia; 6 Department of Internal Medicine and Medical Sciences (DiMI), School of Medicine, University of Genova, Genova, Italy; 7 Department of Medical Oncology, U.O.C Clinica di Oncologia Medica, IRCCS Ospedale Policlinico San Martino, Genova, Italy

**Keywords:** Triple-negative breast cancer, Immunotherapy, Antibody-drug conjugates, Target therapy

## Abstract

Triple-negative breast cancer (TNBC) is a disease with often an aggressive course and a poor prognosis compared to other subtypes of breast cancer. TNBC accounts for approximately 10%–15% of all diagnosed breast cancer cases and represents a high unmet need in the field. Up to just a few years ago, chemotherapy was the only systemic treatment option for this subtype (1). To date, TNBC is considered a heterogeneous disease. One of the existing classifications is based on the analysis of mRNA expression in 587 TNBC cases, in which Lehman et al. proposed six subtypes of TNBC as follows: two basal-like (BL1 and BL2) subtypes, a mesenchymal (M) subtype, a mesenchymal stem-like (MSL) subtype, an immunomodulatory (IM) subtype, and a luminal androgen receptor (LAR) subtype (2). Later studies have demonstrated that the IM and MSL subtypes do not correlate with independent subtypes but reflect background expression by dense infiltration of tumor-infiltrating lymphocytes (TILs) or stromal cells. According to this finding, the classification of TNBC has been revised into the following four subtypes: basal 1, basal 2, LAR, and mesenchymal subtypes (3). Over the last years, several new strategies have been investigated for the treatment of patients with TNBC. Among them, immunotherapy, antibody drug conjugates, new chemotherapy agents, and targeted therapy have been and are currently being developed. The present article aims to provide an updated overview on the different treatment options that are now available or are still under investigation for patients with TNBC.

## Introduction

Triple-negative breast cancer (TNBC) is a disease with often an aggressive course and a poor prognosis compared to other subtypes of breast cancer. TNBC accounts for approximately 10%–15% of all diagnosed breast cancer cases and represents a high unmet need in the field. Up to just a few years ago, chemotherapy was the only systemic treatment option for this subtype [1]. To date, TNBC is considered a heterogeneous disease. One of the existing classifications is based on the analysis of mRNA expression in 587 TNBC cases, in which Lehman et al. proposed six subtypes of TNBC as follows: two basal-like (BL1 and BL2) subtypes, a mesenchymal (M) subtype, a mesenchymal stem-like (MSL) subtype, an immunomodulatory (IM) subtype, and a luminal androgen receptor (LAR) subtype [2]. Later studies have demonstrated that the IM and MSL subtypes do not correlate with independent subtypes but reflect background expression by dense infiltration of tumor-infiltrating lymphocytes (TILs) or stromal cells. According to this finding, the classification of TNBC has been revised into the following four subtypes: basal 1, basal 2, LAR, and mesenchymal subtypes [3]. Over the last years, several new strategies have been investigated for the treatment of patients with TNBC. Among them, immunotherapy, antibody drug conjugates, new chemotherapy agents, and targeted therapy have been and are currently being developed. The present article aims to provide an updated overview on the different treatment options that are now available or are still under investigation for patients with TNBC.

## Immunotherapy

Immune checkpoint inhibitors (ICIs) have become the standard of care for many different tumors in recent years, including melanoma [4], lung cancer [5], urological tumors [6,7], and Hodgkin’s lymphoma [8]. The rationale for immunotherapy research in TNBC is due to the enrichment of TILs. In addition, the expression of PD-L1 on the immune cells of the tumor infiltrate is also relatively frequent [9,10].

### Atezolizumab

Atezolizumab is a fully humanized IgG1 antibody that binds to the PD-L1 receptor. The first evidence for the efficacy of CPIs in TNBC originates from a phase 1 study of atezolizumab monotherapy, which included 116 patients, of whom 91 (78%) had PD-L1 expression ≥1% on immune cells (ICs) (using Ventana SP142 diagnostic antibody). The objective response rate (ORR) was higher in patients who were treated with atezolizumab as the first line treatment (24%) compared to those treated with atezolizumab as the second and subsequent lines of treatment (6%). In addition, patients who had PD-L1 expression ≥1% on ICs had a higher ORR and longer overall survival (OS) than those with PD-L1 expression lower than 1% on ICs (12% *vs*. 0% for 10.1 months *vs*. 6 months, respectively) [11]. Another phase 1b study has investigated the safety and efficacy of the combination of atezolizumab and nab-paclitaxel in various solid tumors. In total, 33 patients with TNBC who received at least two prior lines of systemic therapy were included; the ORR was 39.4%, while the disease control rate (DCR) was 51.5%. In addition, grades 3–4 adverse events were reported in 73% of patients. Further, the median progression-free survival (PFS) was 5.5 months, while the OS was 14.7 months [12].

These results led to the design of the IMpassion130 phase 3 clinical trial, which compared atezolizumab in combination with nab-paclitaxel to placebo with nab-paclitaxel for the first-line treatment of patients with metastatic TNBC (mTNBC). The study coprimary endpoints were PFS and OS, and it hierarchically tested first in the intent-to-treat (ITT) population and then in the PD-L1-positive subgroup (IC >1%, Ventana SP142 antibody). The study did not include patients with relapse within 12 months since the completion of (neo)adjuvant chemotherapy for the treatment of stage 1–3 TNBC. The addition of atezolizumab resulted in longer PFS in the ITT population (7.2 *vs*. 5.5 months, HR 0.80; 95% confidence interval [CI], 0.69 to 0.92; *p* = 0.002) and in the PD-L1-positive subgroup (7.5 *vs*. 5.0 months, HR 0.62; 95% CI, 0.49 to 0.78; *p* < 0.001) [13]. There was no significant difference in OS between both arms in the ITT population, which hampered formal statistical analysis of OS outcomes in the PD-L1-positive subgroup. In the exploratory final analysis, the OS in the PD-L1-positive population was numerically higher with the addition of atezolizumab (25.4 *vs*. 17.9 months, HR 0.67; 95% CI, 0.45 to 0.86) [14].

Considering the greater utilization of paclitaxel in the treatment of breast cancer and the lack of access to nab-paclitaxel in a significant number of countries, the IMpassion131 study was designed. This study had a similar clinical design to IMpassion130 and included the same patient population. However, instead of nab-paclitaxel as the backbone chemotherapy, paclitaxel was used in the IMpassion131 study. In addition, the primary endpoints of the study were different. In the Impassion131 study, the PFS in the PD-L1-positive population and the PFS in the ITT population were tested hierarchically, and the secondary endpoint was OS. In this study, the addition of atezolizumab did not contribute to a longer PFS in both the PD-L1-positive and ITT populations. In the PD-L1-positive population, the median PFS was 6.0 *vs*. 5.7 months (HR 0.82; 95% CI 0.60–1.12; *p* = 0.20) for atezolizumab-paclitaxel and placebo-paclitaxel, respectively. The final OS results demonstrated no difference between the treatment arms (22.1 *vs*. 28.3 months in the PD-L1-positive population; HR 1.11; 95% CI 0.76–1.64), and there were consistent results in the ITT population [15].

The statistical design of the IMpassion130 study hampered proper interpretation of the associated OS benefit with atezolizumab in combination with nab-paclitaxel, and the IMpassion131 study was underpowered for OS analysis in the PD-L1-positive subgroup. Nevertheless, there are numerous hypotheses and theories as to why the results of the IMpassion130 and IMpassion131 studies differ. Although the patient populations were similar based on traditional parameters, a significant imbalance of TNBC subtypes between arms may exist with prognostic and therapeutic implications. In this regard, the PD-L1-positive subgroup of the control arm in the IMpassion131 trial had an OS of 28.3 months, which was longer than what has been previously observed in trials of mTNBC [15,16]. Another potential, although less likely, explanation is the use of corticosteroids for the premedication of paclitaxel [17]. Based on the results of the IMpassion131 study, the Roche company withdrew the registration from the FDA in August 2021, while the EMA revised the registration for the use of atezolizumab only with nab-paclitaxel.

Atezolizumab has also been investigated in studies of early TNBC (eTNBC). The NeoTRIPaPDL1 Michelangelo study investigated neoadjuvant therapy in combination with carboplatin and nab-paclitaxel with or without atezolizumab. Both regimens were administered for a total of eight cycles followed by surgery and four cycles of anthracycline-based therapy. The primary endpoint was event-free survival five years after randomization of the last patient, while the secondary endpoint was pathologic complete response (pCR) rates in the breast and axillary lymph nodes. The NeoTRIPaPDL1 Michelangelo study included 280 female patients. The addition of atezolizumab did not significantly improve the pCR rate compared to chemotherapy alone (43.5% *vs*. 40.8%) [18]. Updated analysis has reported that patients with higher PD-L1 expression have a greater benefit of additional atezolizumab. In patients with an IC score of 2–3 (expression greater than 5% IC, Ventana SP142), the pCR rates were 86.9% *vs*. 72%, respectively, while in patients with PD-L1 expression ranging from 1%–5% IC, the pCR rates were 56.2% *vs*. 44% in the placebo group. In the PD-L1 negative subgroup, patients treated with chemotherapy without the addition of atezolizumab achieved a higher rate of pCR compared to that of the placebo group (41.1% *vs*. 35.1%). In addition, patients in the chemotherapy arm had a higher rate of TILs, which may have contributed to the fact that there was no statistically significant difference in the pCR rates in the ITT population [52.3% *vs*. 47.7% in favor of the atezolizumab arm (*p* = 0.46)] [19].

IMpassion031 is a trial comparing atezolizumab *vs*. placebo as an addition to neoadjuvant 12 weekly cycles of nab-paclitaxel followed by dose-dense anthracycline-based chemotherapy every 2 weeks for a total of 8 weeks followed by surgery. The primary endpoints of the study were pCR in the ITT population and the PD-L1-positive population. The trial randomized 333 patients, of whom 152 were PD-L1 positive. Higher pCR rates were observed for the atezolizumab arms in the ITT population (58% *vs*. 41%; rate difference 17%, 95% CI 6–27; one-sided *p* = 0.0044) and in the PD-L1-positive population (69% *vs*. 49%; rate difference of 20%, 95% CI 4–35; one-sided *p* = 0.021) [20]. Survival results from this study are pending.

### Pembrolizumab

Pembrolizumab is a humanized antibody targeting PD-1. The first results of pembrolizumab activity in TNBC were published as part of the KEYNOTE-012 phase 1b trial. The study included patients with PD-L1-positive (stromal and tumor cell positivity ≥1%) gastric, urothelial, and head and neck cancers. A total of 27 patients with mTNBC were tested. The ORR was 18.5%, and the duration of response was not reached (15–47.3 weeks) [21,22]. The KEYNOTE-086 phase 2 study had two cohorts as follows: cohort A included previously treated mTNBC patients, and cohort B included systemic therapy-naïve metastatic TNBC patients with PD-L1-positive tumors [combined positive score (CPS score) ≥1, DAKO 22C3 antibody]. Cohort A included 170 patients, and 61.8% of whom had PD-L1-positive tumors. Moreover, 43.5% of patients had previously received three or more lines of treatment. The ORR was 5.3% and 5.7% in the total and PD-L1-positive populations, respectively. The median PFS was 2.0 months, and the PFS at 6 months was 14.9%. In addition, the OS at 6 months was 69.1%, and the median OS was 9.0 months. Grade 3–4 adverse events were reported in 12.9% of cases [22]. Cohort B included 84 patients, and 86.9% of whom received prior (neo)adjuvant chemotherapy. Eight patients (9.5%) experienced grade 3 adverse events, and no patient had a grade 4 adverse event. The ORR was 21.4%, and the median DoR was 10.4 months. Moreover, the PFS was 2.1, and the OS was 18 months [23,24].

The first phase 3 study investigating pembrolizumab in mTNBC was the KEYNOTE-119 trial. Patients who were previously treated with 1–2 lines of systemic therapy for mTNBC were randomized to pembrolizumab or chemotherapy selected by the investigators as follows: capecitabine, eribulin, vinorelbine, or gemcitabine. The primary endpoints were OS in participants with a PD-L1 CPS score of 10 or higher followed by a CPS score ≥1 in the ITT population by hierarchical testing. The trial included 1098 participants who were randomized 1:1. The study did not demonstrate the superiority of pembrolizumab over chemotherapy. The median OS values in the pembrolizumab *vs*. chemotherapy arms were as follows: 12.7 *vs*. 11.6 months (HR 0.78; 95% CI 0.57–1.06; log-rank *p* = 0.057) in patients with CPS ≥10; 10.7 *vs*. 10.2 months in patients with CPS ≥1 (HR 0.86; 95% CI 0.69–1.06; log-rank *p* = 0.073); and 9.9 *vs*. 10.8 months in the ITT population. An exploratory subgroup analysis in patients with CPS ≥20 suggested an OS benefit of pembrolizumab over single-agent CT of the investigator’s choice. However, the role of pembrolizumab as a single agent in this setting has not been further investigated, and pembrolizumab as a single agent has only been approved by the FDA for TNBC with high TMB or MS instability [25].

In contrast to the KEYNOTE-119 study, the KEYNOTE-355 first-line mTNBC study established pembrolizumab in combination with chemotherapy as a new standard of care for mTNBC in patients with PD-L1-positive (CPS ≥10) tumors. This phase 3 study compared the addition of pembrolizumab to first-line chemotherapy according to the investigator’s choice as follows: paclitaxel, nab-paclitaxel, or gemcitabine/carboplatin combination. The primary study endpoints were PFS and OS in the PD-L1 CPS ≥10, PD-L1 CPS ≥1, and ITT populations as tested hierarchically. In the CPS ≥10 subgroup, progression-free survival was significantly longer for patients in the chemotherapy/pembrolizumab arm (median PFS 9.7 *vs*. 5.6 months HR for progression or death 0.65, 95% CI 0.49–0.86; one-sided *p* = 0.0012). In the subgroup of patients with CPS ≥1, no statistically significant difference in PFS was achieved, precluding formal analysis of PFS in the ITT population. Among patients with a CPS of 1 or more, the median PFS was 7.6 *vs*. 5.6 months [HR 0.74; 0.61–0.90; one-sided *p* = 0.0014 (not significant)] and 7.5 *vs*. 5.6 months [HR 0.82; 0.69–0.97 (not tested)] in the ITT population. Based on these results, the FDA approved pembrolizumab in combination with chemotherapy for the CPS ≥10 subgroup of patients in November 2020 [26]. At ESMO 2021, the final analysis of the KEYNOTE-355 study was presented, indicating that the addition of pembrolizumab to chemotherapy is also associated with an improvement in OS exclusively in the CPS ≥10 subgroup (23 *vs*. 16.1 months OS; HR 0.73, *p* = 0.0093) [27].

Pembrolizumab has also been investigated in early and locoregionally advanced TNBC. After promising results of the KEYNOTE-173 [28] and I-SPY2 [29] trials, the KEYNOTE-522 phase 3 study was designed to compare the addition of pembrolizumab to standard neoadjuvant chemotherapy in patients with either cT1cN1-2 or cT2-4N0-2. Patients were randomized to receive neoadjuvant chemotherapy with paclitaxel and carboplatin followed by four cycles of anthracycline-based chemotherapy given every 3 weeks plus pembrolizumab or placebo. Following surgery, patients were treated with up to nine additional cycles of pembrolizumab or placebo. The primary endpoints were the pCR rate and event-free survival (EFS). Primary pCR analysis, which was performed in the first 602 randomized patients, demonstrated a significantly higher pCR rate in the pembrolizumab arm (64.8% *vs*. 51.2%, *p* = 0.00055) [30]. At the third EFS interim analysis, the EFS benefit from pembrolizumab crossed the predefined significance boundary (36-months EFS 84.5% *vs*. 76.8%, HR 0.63, *p* < 0.001) [31]. Subgroup analysis suggested a larger pCR benefit from pembrolizumab in patients in the node-positive group, but the impact from pembrolizumab on EFS was similar in node-positive and node-negative patients. In patients who had achieved a pCR, the EFS at 36 months was high (94.4% *vs*. 92.5%, in the pembrolizumab arm compared to placebo, respectively) compared to the EFS in the group of patients who did not achieve a pCR (67.4% *vs*. 56.8%, in the pembrolizumab group compared to placebo, respectively). The addition of pembrolizumab demonstrated a benefit independent of PD-L1 status with pCR rates being higher in PD-L1-positive patients in both arms [30,31].

### Durvalumab

Durvalumab is an anti-PD-L1 antibody that was investigated as part of the SAFIR 02-BREAST IMMUNO trial, which included patients with metastatic HER2-negative HR+ or triple-negative breast cancer. Patients who did not progress after 6–8 cycles of chemotherapy were randomized to durvalumab or maintenance chemotherapy. The study included 199 patients. Overall, durvalumab did not prolong PFS (HR 1.4; 95% CI: 1.00–1.96; *p* = 0.047) or OS (HR 0.84, 95% CI: 0.54–1.29; *p* = 0.423). However, in an exploratory analysis of the TNBC cohort (n = 82), durvalumab prolonged OS (HR 0.54; 95% CI: 0.30–0.97; *p* = 0.0377). An even greater benefit was achieved in TNBC patients with a PD-L1 expression (HR of death of 0.37; 95% CI: 0.12–1.13) [32]. Because this was an exploratory analysis, the results served as a basis to generate hypotheses for other trials with durvalumab.

Durvalumab was investigated in early TNBC in the GeparNuevo phase 2 study. In this trial, patients with tumors sized at least 2 cm were randomized to durvalumab/placebo with neoadjuvant chemotherapy, including nab-paclitaxel, followed by an epirubicin/cyclophosphamide combination. The GeparNuevo study did not include administration of ICI in the adjuvant treatment part. The initial design of the study included a window, in which patients received durvalumab/placebo monotherapy for two cycles followed by chemotherapy. Since this approach led to a delay in starting chemotherapy, the regulatory bodies canceled the window of the trial, and the inclusion of patients continued with classic randomization. Durvalumab numerically increased the rate of pCR (53.4% *vs*. 44.2%), but the difference was not statistically significant. Subgroup analysis of patients included in the window part of the study showed a statistically significant difference in favor of the durvalumab group (61% *vs*. 41.1%, OR 2.22, *p* = 0.035) [33]. After three years of follow-up, durvalumab significantly prolonged invasive disease-free survival (IDFS) (84.9% *vs*. 76.9%, HR 0.54, *p* = 0.0559), distant DFS (DDFS) (91.4% *vs*. 79.5%, HR 0.37, *p* = 0.0148), and OS (95.1% *vs*. 83.1% HR 0.26, *p* = 0.0076) [34]. Table 1 summarizes immune checkpoint inhibitors in TNBC.

**Table 1:** Immune checkpoint inhibitors in triple-negative breast cancer

Drug [Ref.]	Target	No. of patients	Study design	Study phase	Results
Atezolizumab	PD-L1				
[13,14]		902	Untreated metastatic TNBC or relapse after more than 12 months of neoadjuvant/adjuvant therapy atezo+nab-paclitaxel *vs*. placebo+nab-paclitaxel	3	PFS (ITT) 7.2 *vs*. 5.5 months, HR 0.80, 95% CI 0.69–0.92 *p* = 0.002 PFS (PD-L1 positive) 7.5 *vs*. 5.0 months, HR 0.62, 95% CI 0.49–0.78; *p* < 0.001 OS (ITT) 21.3 *vs*. 17.6 months, HR 0.84, *p* = 0.08 OS (PD-L1 positive) 25.4 *vs*. 17.9 months, HR 0.67 95% CI 0.45–0.86
[15]		651	Untreated metastatic TNBC or relapse after more than 12 months of neoadjuvant/adjuvant therapy atezo+paclitaxel *vs*. placebo+paclitaxel	3	PFS (PD-L1 positive) 6.0 *vs*. 5.7 months, HR 0.82 95% CI 0.60–1.12 *p* = 0.20 PFS (ITT) 5.7 *vs*. 5.6 months, HR 0.86 95% CI 0.70–1.05 OS (PD-L1 positive) 22.1 *vs*. 28.3 months, HR 1.11 95% CI 0.76–1.64 OS (ITT) 19.2 *vs*. 22.8 months, HR 1.12 95% CI 0.88–1.43
[18,19]		280	Neoadjuvant TNBC carbo+nab-paclitaxel+atezo *vs*. carbo+nab-paclitaxel+placebo Surgery Adjuvant anthracyclines	3	pCR (ITT) 43.5% *vs*. 40.8% pCR (PD-L1 positive IC score 2–3) 86.9% *vs*. 72% pCR (PD-L1 positive IC score 1) 56.2% *vs*. 44% pCR (PD-L1 negative) 35.09% *vs*. 41.07%
[20]		333	Neoadjuvant TNBC atezo+nab-paclitaxel+anthracyclines *vs*. placebo+nab-paclitaxel+anthracyclines Surgery	3	pCR (ITT) 58% *vs*. 41%, *p* = 0.004 pCR (PD-L1 positive) 69% *vs*. 49%, *p* = 0.021
Pembrolizumab	PD-1				
[26,27,35]		847	Untreated metastatic TNBC or relapse more than 6 months after neoadjuvant/adjuvant chemotherapy pembro+paclitaxel or nab-paclitaxel or gem/carbo *vs*. placebo+paclitaxel or nab-paclitaxel or gem/carbo	3	PFS (PD-L1 positive CPS ≥10) 9.7 *vs*. 5.6 months, HR 0.66 95% CI 0.50–0.88 PFS (PD-L1 positive CPS 1–9) 7.6 *vs*. 5.6 months, HR 0.75 95% CI 0.62–0.91 PFS (ITT) 7.5 *vs*. 5.6, HR 0.82 95% CI 0.70–0.98 OS (PD-L1 positive CPS ≥10) 23.0 *vs*. 16.1, HR 0.73 95% CI 0.55–0.95 OS (PD-L1 positive CPS 1–9) 17.6 *vs*. 16.0, HR 0.86 95% CI 0.72–1.04 OS (ITT) 17.2 *vs*. 15.5, HR 0.89 95% CI 0.76–1.05
[30,31]		602	Neoadjuvant TNBC pembro+paclitaxel+carbo+anthracyclines *vs*. placebo+paclitaxel+carbo+anthracyclines Surgery Adjuvant pembro *vs*. placebo	3	pCR (ITT) 64.8% *vs*. 51.2%, 95% CI 5.4–21.8 *p* = 0.00055 EFS (ITT) EFS 84.5% *vs*. 76.8%, HR 0.63, 95% CI 0.43–0.93 *p* < 0.001 EFS (pCR) 94.4% *vs*. 92.5% EFS (non-pCR) 67.4% *vs*. 56.8%
Durvalumab[33,34]	PD-L1	174	Neoadjuvant TNBC durvalumab or placebo (2 cycles) durva+nab-paclitaxel+antracyclines *vs*. placebo+nab-paclitaxel+antracyclines (first part of study) durva+nab-paclitaxel+antracyclines *vs*. placebo+nab-paclitaxel+antracyclines (second part of study)	2	pCR (first part) 61% *vs*. 41.1%, OR 2.22, 95% CI 1.06–4.64 *p* = 0.035 pCR (second part) 53.4% *vs*. 44.2% IDFS 84.9% *vs*. 76.9%, HR 0.54, 95% CI 0.16–0.27; *p* = 0.0559 DDFS 91.4% *vs*. 79.5%, HR 0.37, 95% CI 0.11–0.69; *p* = 0.0148 OS 95.1% *vs*. 83.1% HR 0.26, 95% CI 0.09–0.81; *p* = 0.0076

Note: TNBC-triple negative breast cancer, atezo-atezolizumab, PFS-progresion free-survuval, OS-overall survival, EFS-event free-survuval, IDFS-invasive disease free-survuval, DDFS-distant disease free-survival, pCR-pathological complete response, pembro-pembolizumab, durva-durvalumab, gem-gemcitabine, carbo-carboplatin.

### Open clinical questions in immune checkpoint inhibitor therapy for TNBC

There are several open questions regarding the treatment of TNBC with ICIs. First, PD-L1 expression is confirmed as a predictive biomarker for benefit from the addition of ICI to chemotherapy in the metastatic TNBC setting. Studies with ICIs have used different diagnostic assays, different cells counted in the score, and different cutoffs for PD-L1 positivity. In the IMpassion130 study, the Ventana SP142 diagnostic antibody was used, and a cutoff of ≥1% positivity on immune cells was adopted [13]. However, in the KEYNOTE-355 study with pembrolizumab, the DAKO 22C3 antibody was used, and the benefit of pembrolizumab was demonstrated only in patients with CPS ≥10 [27,35]. Rugo et al. performed a translational analysis of the IMpassion130 study to examine the concordance of the following three different assays for determining PD-L1 positivity, all with a cut off value of 1% on immune cells (ICs): Ventana SP142 (which was initially used in the study), Ventana SP263, and DAKO22C3. The PD-L1 positivity was 46.4%, 74.9%, and 73.1% with the use of the SP142, SP263 and 22C3 antibodies, respectively. Almost all cases of SP142 positivity were accompanied by positivity of the other two antibodies. However, 29.6% of SP263 positivity and 29% of 22C3 positivity were shown to be SP142 negative. The most important conclusion of this analysis was that only patients with SP142 positivity benefit from the addition of atezolizumab to nab-paclitaxel [36]. The clinical value of the results of this analysis was reduced by the later knowledge that only those patients with a CPS score ≥10 benefit from the addition of pembrolizumab to chemotherapy in first-line mTNBC treatment. To date, there is no analysis comparing IC ≥1 SP142 and CPS ≥10 22C3. Harmonizing diagnostic antibodies in mTNBC may open the possibility of using atezolizumab for PD-L1 22C3 antibody positivity or pembrolizumab for SP142 antibody positivity. Until then, it has been recommended that for each of the two drugs, PD-L1 positivity is determined according to the diagnostic antibody used in the registration study [36].

Another open question is to understand why patients appear to benefit more from ICIs in the neoadjuvant setting compared to metastatic disease and why this benefit in the early setting is not dependent on PD-L1 expression. There are several possible explanations of this phenomenon. Patients in the early stages of the disease are more immunocompetent, and the host’s immune system reacts more easily to foreign antigens being less immunocompromised by previous treatments. Another reason is the presence of different cells in the tumor microenvironment. Namely, in regard to early breast cancer, the cells that lead to exhaustion of effector T lymphocytes and the reduction of the overall response are less represented [37–39].

The third question is to understand how to treat patients with early relapse within 12 months of completing (neo)adjuvant chemotherapy. In the IMpassion130 study, a disease-free interval (DFI) of less than 12 months was an exclusion criterion [13], while in the KEYNOTE-355 study, the exclusion criterion was DFI <6 months. In the subgroup analysis of patients with a shorter DFI of 6–12 months, the results suggested less benefit from the addition of pembrolizumab [35]. The answer to this question is expected from the IMpassion132 study, which compares the addition of atezolizumab to the combination of gemcitabine/carboplatin or capecitabine (according to the physicians’ choice) in patients who relapsed within one year from the end of (neo)adjuvant chemotherapy [40]. In addition, questions regarding treatment with ICI after ICI therapy in the early stage of TNBC remain unanswered. It is unclear whether there can be an influence of DFI or possibly a change in CP. The optimal duration of CPI therapy is a topic of debate as well, especially when it comes to patients who achieve pCR after NAT. According to the treatment regimen in the KEYNOTE-522 study, guidelines recommend adjuvant therapy with pembrolizumab irrespective of pCR [31]. However, the results of the GeparNuevo study raise the hypothesis that patients who achieve pCR may have no or limited benefit from immunotherapy administration in the adjuvant treatment part [34]. Prospective studies are needed to answer the question of whether patients with TNBC achieving pCR with immunotherapy can be safely spared from further adjuvant systemic treatment.

Apart from the IMpassion132 study [40], which will clarify the role of CPI in early relapsing patients, several other ongoing studies will answer questions about the role of CPI in the treatment of TNBC. The final analyses of the NeoTRIPaPDL1 Michelangelo [18], IMpassion031 [20], and IMpassion030/ALEXANDRA [41] studies will reveal whether there is a role for atezolizumab in early TNBC. We expect the A_BRAVE study to define the role of avelumab in the postneoadjuvant setting [42], and the SWOG S1418 study [43] will further investigate the role of pembrolizumab in the adjuvant setting. Numerous studies in metastatic and early TNBC have been designed to identify the optimal role and position of ICI in the treatment of this disease [17].

## Antibody-Drug Conjugates

In recent years, new effective drugs with more potency and specificity than traditional drugs to tumor surface proteins have been developed. These new drugs are antibody‒drug conjugates, in which a monoclonal antibody against the cancer cell target protein is conjugated to a cytotoxic agent. The first ADCs approved for clinical use were ado-trastuzumab emtansine and brentuximab vedotin, which were followed by many others aiming to identify better targets, more effective cytotoxic drugs, and more sophisticated linker technology [44].

### Sacituzumab-govitecan

Sacizutumab-govitecan (SG) is an antibody‒drug conjugate consisting of a humanized monoclonal antibody that targets trophoblastic cell-surface antigen 2 (TROP2). The SN-38 payload, an active metabolite of irinotecan and inhibitor of DNA topoisomerase I, is conjugated to the antibody with a pH-sensitive cleavable linker. TROP2 is a transmembrane calcium signal transducer involved in several pro-oncogenic signaling pathways and is expressed in a wide variety of epithelial tumors, including TNBC [45–47]. The IMMU-132-01 phase 1/2 study examined the safety and efficacy of SG in various advanced solid tumors. The TNBC cohort included 108 patients who had been previously treated with at least two lines of systemic therapy for metastatic disease. The median number of prior therapies was 3 (2–10). The most common grade 3 or 4 adverse effects were anemia and neutropenia, while 9.3% of patients had febrile neutropenia. The objective response rate was 33.3% (three patients had CR, 33 PR). Moreover, the median PFS, OS, and CBR were 5.5 months, 13 months, and 45.4%, respectively [48].

The ASCENT phase 3 study [49] compared SG to single-agent chemotherapy of investigator’s choice (capecitabine, vinorelbine, eribulin, or gemcitabine) in patients with TNBC who had been previously treated with at least two lines of chemotherapy for advanced disease or at least one line for metastatic disease if relapse occurred within one year of (neo)adjuvant chemotherapy. The primary endpoint was PFS without baseline brain metastases. In the study, 468 patients were randomized, and the median age was 54 years. Moreover, all patients were previously treated with taxanes. The median PFS in the SG arm was 5.6 months *vs*. 1.7 months in the control arm (HR 0.41; 95% CI, 0.32 to 0.52; *p* < 0.001). Overall survival, which was the key secondary endpoint of the study, was also longer in the SG arm (12.1 *vs*. 6.7; HR 0.48; 95% CI: 0.38 to 0.59; *p* < 0.001). In addition, the ORR on SG was 35% *vs*. 5% in control patients. Grade 3 adverse events and higher were more frequent in the SG group (51% *vs*. 33%). The most common toxicities were leukopenia (10% *vs*. 5%), anemia (8% *vs*. 5%), diarrhea (10% *vs*. <1%), and febrile neutropenia (6% *vs*. 2%). Based on the ASCENT study, SG is the recommended standard of care in pretreated patients with mTNBC from second-line treatment onward [50,51]. The most common adverse effect of SG parallel those of chemotherapy and although frequent, are highly manageable with timely and proactive interventions [52].

Biomarker analyses report of the ASCENT trial has evaluated the link between Trop-2 expression and germline BRCA1/2 status and survival outcomes in patients treated with sacituzumab govitecan. The analyses report 80% of the patients to have high/medium tumor Trop-2 expression. Benefit of these patients was similar to ITT population and was observed regardless BRCA1/2 status. Median OS and ORR were numerically higher in SG treatment group in all the Trop-2 expression subgroups compared to chemotherapy [53].

SG is being investigated in the following studies: first-line treatment of mTNBC *vs*. investigator’s choice in PD-L1-negative tumors previously treated with ICIs in the early setting that express PD-L1 (ASCENT 03 trial; NCT05382299) [54]; in combination with pembrolizumab *vs*. investigator’s choice in PD-L1-positive tumors (ACENT 04 trial; NCT05382286) [55]; in combination with pembrolizumab or alone in mTNBC that are PD-L1 negative (NCT04468061) [56]; in combination with talazoparib in mTNBC (NCT04039230) [57]; as well as in the early setting after standard NACT against resistant residual disease (SASCIA trial; NCT04595565) [58], and multi arm phase II study NeoSTAR investigating SG in neoadjuvant setting (NCT04230109) [59].

### Trastuzumab-deruxtecan (T-DXd)

The breakthrough in development of new ADCs for HER2-low BC revolutionized the treatment landscape in breast cancer.

Trastuzumab-deruxtecan (T-Dxd) consists of a humanized anti-HER2 monoclonal antibody linked to a topoisomerase I inhibitor payload through a tetrapeptide-based cleavable linker [60]. Based on the DESTINYBreast-03 study, T-DXd has been registered for second-line treatment of metastatic HER2-positive breast cancer [61].

In the DESTINYBreast-04 study, T-DXd was compared to chemotherapy of the researcher’s choice in HER2-low advanced breast cancer, i.e., in cancers with immunohistochemical expression of HER2 1+ or 2+ with negative *in situ* hybridization. Patients were previously treated with at least one and maximum two lines of chemotherapy for advanced disease, while patients with HR-positive tumors previously received a CDK4/6 inhibitor. The study planned to include 480 patients with HR+ tumors and 60 patients with TNBC. A total of 58 patients with TNBC were randomized out of the 557 patients included in the trial. The primary endpoint was PFS in HR+ patients, and the key secondary endpoints were PFS in all patients and OS in HR+ of all enrolled patients. PFS, which was the primary endpoint, in HR+ patients was longer than in the T-DXd group (10.1 *vs*. 5.4 months; HR 0.51; 95% CI, 0.40 to 0.64; *p* < 0.001). The overall survival of HR+ patients was also longer in the T-DXd group (23.9 *vs*. 17.5 months; HR 0.64; 95% CI, 0.48 to 0.86; *p* = 0.003). Trastuzumab deruxtecan demonstrated similar safety profile to the safety profile of the patients with HER2 positive BC. Compared to physicians’ choice of chemotherapy, grade 3 or higher adverse events occurred in 52.6% of the patients treated with trastuzumab deruxtecan *vs*. 67.4% of those who received the physician’s choice of chemotherapy. The most common adverse events grade 3 or higher was neutropenia (in 13.7% of patients on TDX-d *vs*. 40.7% on chemotherapy), anemia (in 8.1% *vs*. 4.7%), and fatigue (7.5% *vs*. 4.7% of patients). Drug-related interstitial lung disease or pneumonitis occurred in 12.1% of the patients who received trastuzumab deruxtecan [62].

In the cohort of 58 patients with HER2-low TNBC, the group receiving T-DXd had a numerically longer PFS (8.5 *vs*. 2.9 months; HR 0.46; 95% CI, 0.24 to 0.89) and OS (18.2 *vs*. 9.9 months, HR 0.48; 95% CI, 0.24 to 0.95) [62]. DESTINY-breast04 established TDX-d as a new standard of care in HER2-low metastatic BC. Based on these results, the FDA approved T-DXd as the first targeted therapy for this mBC subtype [63].

### Datopotamab-deruxtecan (Dato-DXd)

Datopotamab-deruxtecan (Dato-DXd) is an antibody‒drug conjugate targeting TROP2 with deruxtecan as the payload. In the TROPION-PanTumor01 study, 44 patients with TNBC were included. The treatment response was 34%, while the CBR was 77%. In the subgroup of patients (n = 27) who did not receive prior ADCs (with deruxtecan) and TROP2-targeted therapy, the ORR was 53%, while 15% of patients had PD. The most common side effects of Dato-DXd were stomatitis, vomiting, and fatigue. In addition, 18% of patients required dose reduction due to adverse effects, and the treatment was interrupted in 14% of patients [64]. Following these results, the TROPION-Breast02 phase study was designed for locally advanced or metastatic TNBC in which CPI therapy is not indicated. The study compared Dapo-DXd to the investigator’s choice of therapy, and the coprimary endpoints were PFS and OS [65, NCT05374512]. Table 2 summarizes antibody-drug conjugates in TNBC.

**Table 2:** Antibody-drug conjugates in triple-negative breast cancer

	Target	No. of patients	Study design	Study phase	Results
Sacituzumab-govitecan	TROP2				
[49]		902	Previously treated metastatic TNBC SG *vs*. investigator choice of therapy (capecitabine, vinorelbin, eribulin or gemcitabine)	3	PFS 5.6 *vs*. 1.7 months, HR 0.41 95% CI 0.32–1.52; *p* < 0.001. OS 12.1 *vs*. 6.7 mos; *p* < 0.001, HR 0.48 95% CI 0.32–0.52; ORR 35% *vs*. 5%
Trastuzumab-deruxtecan	HER2				
[57]		557 (58 HR-)	Previously treated metastatic HER2 low HR positive and TNBC T-DXd *vs*. investigator choice of therapy (capecitabine, paclitaxel, nab-paclitaxel, eribulin or gemcitabine)	3	PFS (TNBC) 8.5 *vs*. 2.9 mos; HR 0.46 95% CI 0.24–0.89; OS (TNBC) 18.2 *vs*. 9.9 mos, HR 0.48 95% CI 0.24–0.95;

Note: TNBC-triple negative breast cancer, PFS-progresion free-survuval, OS-overall survival, SG-sacituzumab govitecan, T-dxd-Trastuzumab-deruxtecan, HR-hormone receptor.

## Other Targeted Therapies

### PARP inhibitors

Approximately 5–10% of breast cancer cases are associated with germline BRCA1/2 pathogenic variants. However, more than 20% of patients with TNBC are carriers of germline BRCA1/2 pathogenic variants [66]. These patients are candidates for treatment with poly (ADP-ribose) polymerase (PARP) inhibitors [67].

In the OlympiAD study, the PARP inhibitor, olaparib, was compared with investigator’s choice chemotherapy (capecitabine, eribulin, or vinorelbine) in patients with metastatic HER2-negative breast cancer and germline BRCA1/2 pathogenic variants. Patients had previously received a maximum of two lines of chemotherapy for mBC. A total of 302 patients were included (of whom 150 had TNBC), and they were randomized 2:1 in favor of olaparib. PFS, which was the primary endpoint, was longer in the olaparib group (7.0 *vs*. 4.2 months; HR 0.58; 95% CI 0.43 to 0.80; *p* < 0.001). Subgroup analysis of patients with TNBC suggested enhanced benefit from olaparib (HR 0.43) in this population. The most common grade ≥3 adverse events of olaparib were anemia (16.1%) and neutropenia (9.3%). The quality of life reported in this study was better in patients treated with olaparib, while there was no difference in OS [68].

The EMBRACA study compared the PARP inhibitor, talazoparib, with investigator’s choice chemotherapy (capecitabine, vinorelbine, gemcitabine, or eribulin) in patients with germline BRCA1/2 pathogenic variants who had previously received up to two lines of chemotherapy in the metastatic phase of the disease. A total of 431 patients were included (of whom 190 had TNBC), and 287 patients were randomized to talazoparib. The time to disease progression was longer in the talazoparib group (8.6 *vs*. 5.6 months; HR 0.54; 95% CI 0.41 to 0.71; *p* < 0.001), while similar to the OlympiAD study, the OS did not differ. Anemia was the most common side effect of talazoparib along with fatigue and nausea. Unlike the OlympAD study in which olaparib showed benefit in TNBC, the benefit of talazoparib in the EMBRACA study was balanced in both cohorts of HR+ and TNBC patients [69].

The BROCADE3 phase 3 study investigated the veliparib PARP inhibitor *vs*. placebo in combination with paclitaxel/carboplatin chemotherapy. Patients who discontinued chemotherapy before progression could continue veliparib/placebo therapy in maintenance. Additionally, patients who progressed in the placebo group could crossover to veliparib as well. Enrolled patients had HER2-negative metastatic breast cancer with a BRCA1 or BRCA2 germline pathogenic variant, and they were treated with up to two prior lines of chemotherapy for metastatic disease. However, the majority of the patients were not previously treated for metastatic disease. The study included 513 patients with a ratio of 2:1 in the veliparib group. The primary endpoint, PFS, was longer in the veliparib group (14.5 *vs*. 12.6 months, HR 0.71; 95% CI 0.57 to 0.88; *p* = 0.0016). The most common adverse events were neutropenia (81% in the veliparib group *vs*. 84% in the placebo group), anemia (42% *vs*. 30%), and thrombocytopenia (40% *vs*. 28%), while serious adverse events were reported in 34% of patients in the veliparib group *vs*. 29% in the placebo group [70].

Olaparib has also been investigated in a phase 3 trial for high-risk early breast cancer in adjuvant treatment. The study included patients with high-risk HER2-negative early breast cancer and germline BRCA1 or BRCA2 pathogenic variants. To be eligible for the trial, patients with TNBC treated primarily with surgery had to have tumors larger than 2 cm or node-positive disease, while those treated with neoadjuvant therapy were required to have residual tumor in the breast and/or lymph nodes. Patients were randomized to one year of olaparib or placebo therapy. The study included 1836 patients with 82% of patients with TNBC. After a median follow-up of 2.5 years, the three-year invasive disease-free survival (IDFS) in the ITT population was 85.9% and 77.1% in the olaparib and placebo groups, respectively (HR 0.58; 99.5% CI, 0.41 to 0.82; *p* < 0.001). The adverse effects noted were similar to those reported in other studies with PARP inhibitors [71]. At the second interim analysis, after a median follow-up of 3.5 years, olaparib significantly prolonged OS compared to placebo with a 4-year OS 89.8% *vs*. 86.4% (HR 0.68; 98.5% CI 0.47–0.97; *p* = 0.009) in the olaparib group compared to the placebo group [72].

PARP inhibitors have also been investigated as neoadjuvant therapy for patients with HER2-negative breast cancer and germline BRCA pathogenic variants. After promising results of the I-SPY2 trial [73] of veliparib in combination with paclitaxel and carboplatin, the BrighTNess phase III trial was designed. In this trial, the addition of carboplatin, i.e., carboplatin and velibaprib, was investigated in relation to paclitaxel alone. The primary goal of pCR was achieved with the triplet treatment *vs*. paclitaxel alone (53% *vs*. 31%, *p* < 0.0001); however, the addition of veliparib did not affect the pCR rate compared to the combination of carboplatin and paclitaxel with a pCR rate of 58% [74]. In contrast, the PARPi single agent may be promising to avoid chemotherapy in some patients as shown in the NEO TALA phase 3 trial, in which the talazoparib single agent in the neoadjuvant setting in early gBRCA1/2-mutated HER2-negative breast cancer achieved pCR rates comparable to those observed with the combination of anthracycline and taxane-based chemotherapy regimens. The pCR by independent central review in the evaluable population was 45.8% (95% CI 32.0–60.6) [75].

### PI3K-AKT inhibitors

Various genomic alterations, such as PIK3CA or AKT1 activating mutations or PTEN loss can result in PI3K/AKT/mTOR pathway activation. The AKT1, AKT2, and AKT3 proteins are downstream effectors of PI3K and can be targeted by AKT inhibitors [76]. Alpelisib is a selective inhibitor of the catalytic alpha subunit of PI3K (PIK3CA), and it is registered for the treatment of metastatic HR+/HER2-PIK3CA-mutated breast cancer in combination with endocrine therapy [77]. Approximately 50% of TNBC patients have various alterations in the PI3K pathway, making them a potential target for treatment with alpelisib and other PI3K inhibitors [78]. A previous study combining alpelisib with nab-paclitaxel in HER2-negative breast cancer has reported an ORR of 59% and a median PFS of 8.7 months. In this study, 30% of patients had TNBC [79]. Based on these results, the EPIK-B3 study was designed to investigate the addition of alpelisib to chemotherapy in patients with advanced TNBC and PIK3CA mutations or PTEN loss [80, NCT04251533].

The AKT inhibitors, ipatasertib and capivasertib, have also been investigated in two mTNBC phase II trials. The LOTUS study is a randomized trial that compared the combination of the ipatasertib AKT inhibitor *vs*. placebo in combination with paclitaxel in patients with metastatic TNBC. The primary endpoint was PFS in the ITT and immunohistochemical PTEN-low populations. The study included 124 patients. The median PFS was 6.2 in the ipatasertib group compared to 4.9 months in the placebo group (HR 0.6, 95% CI 0.37–0.98; *p* = 0.037). The median PFS was 6.2 months in the group with PTEN-low tumors compared to 3.7 months in the group with ipatasertib *vs*. placebo (HR 0.59, 95% CI 0.26–1.32, *p* = 0.18). The most common side effect of ipatasertib was diarrhea, which occurred in 23% of patients [81].

Another phase 2 study investigating ipatasertib was the FAIRLANE study, which examined the pCR rate in patients with TNBC if ipatasertib was added to standard neoadjuvant chemotherapy. The primary objective was pCR in the ITT and PTEN-low populations, and the secondary objective was the pCR rate in tumors with PI3KA/AKT1/PTEN alterations. The pCR rates were numerically higher in the ipatasertib group (17%) compared to the ITT population (13%). In addition, the pCR rates were 16% with ipatasertib *vs*. 13% with placebo in the PTEN-low population, and 18% *vs*. 12% in the population with PI3KA/AKT1/PTEN-altered tumors [82].

Based on the results of the LOTUS study, the IPATunity phase 3 study was launched. In cohort A of this study, the addition of ipatasertib to paclitaxel was investigated with the control arm receiving placebo with paclitaxel. There was no difference in PFS in the ipatasertib group compared to the placebo group (7.4 *vs*. 6.1 months) in patients with PI3KA/AKT1/PTEN alterations (HR 1.02; 95% CI 0.71–1.45). Similar percentages of patients in both arms had grade ≥3 adverse events (46% *vs*. 44% in the ipatasertib group compared to placebo group, respectively) and toxicities leading to discontinuation of treatment (14% *vs*. 15%, respectively). Adverse events leading to dose reduction were more common with ipatasertib (35% *vs*. 14%). Diarrhea (80% *vs*. 31%; grade ≥3 9% *vs*. 2%), alopecia (46% *vs*. 44%), and nausea (36% *vs*. 23%) were the most common adverse events [83]. Despite the results of the preclinical models and the positive results of a phase 2 trial, there are numerous explanations why this trial reported negative results. The most likely explanation is that there is a large number of alterations affecting this signaling pathway; however, not all of them have functional effects and thus are not affected by AKT inhibition [78].

Capivasertib is an oral AKT inhibitor. The PAKT study compared the addition of capivasertib to paclitaxel in the first-line treatment of mTNBC. The primary objective was PFS in the ITT population, and the secondary objectives were OS in the ITT population and PFS and OS in patients with PI3KA/AKT1/PTEN alterations. The median PFS was 5.9 months in the ipatasertib group and 4.2 months in the placebo group (HR 0.74; 95% CI 0.50 to 1.08; *p* = 0.06). The OS was longer in the ipatasertib group (19.1 *vs*. 12.6 months, HR 0.61; 95% CI, 0.37 to 0.99; *p* = 0.04). In addition, the PFS was significantly longer in the small group (n = 28) with PI3KA/AKT1/PTEN alterations (9.3 with capivasertib plus paclitaxel *vs*. 3.7 months with placebo plus paclitaxel; HR 0.30; 95% CI, 0.11 to 0.79; *p* = 0.01). The most common grade ≥3 adverse event in the capivasertib group was diarrhea (13% *vs*. 1% in the placebo group) [84]. Capivasertib demonstrated efficacy as a monotherapy in the BAY-131-Y study in patients with AKT817K mutations [85]. A CAPItello-29 phase 3 study [86, NCT03997123] with capivasertib is ongoing.

### Targeting the androgen receptor (AR)

Among the molecular subtypes of TNBC, the luminal androgen receptor (LAR) is characterized by a gene expression pattern similar to that of luminal cancers. Compared to other TNBC subtypes, LAR TNBC has high expression of androgen receptor according to IHC analysis, and it is enriched in activating PIK3CA or AKT1 mutations [2,87]. The clinical benefit of single-agent antiandrogens in unselected TNBC or in biomarker-driven subgroups is defined by AR IHC. The clinical benefit rate with bicalumide or enzalutamide ranges from 19% to 35% [88–90]. Several important studies to unravel the role of AR-blockade are underway. One of these is a phase 3 trial comparing the addition of bicalutamide to chemotherapy in AR+ mTNBC [91, NCT03055312]. In addition, similar to luminal carcinomas, PI3KA/AKT inhibitors are being investigated in LAR subtypes of TNBC. In the TBCRC 032 phase 2/3 study, the PI3KA inhibitor, taselisib, was combined with the AR-blocker, enzalutamide. The clinical benefit rate in the LAR subtype was 75% compared to 12.5% in non-LAR tumors [92]. A study examining alpelisib with enzalutamide is ongoing [93, NCT03207529], and a study combining the CDK4/6 inhibitor, palbociclib, with enzalutamide achieved a median 6-month PFS of 33% in the primary analysis [94].

### Trilaciclib

CDK 4/6 inhibitors are the standard of care for metastatic HR+/HER2-breast cancer [50,51]. Trilaciclib is an intravenous CDK4/6 inhibitor that has been investigated in mTNBC in combination with gemcitabine/carboplatin chemotherapy in second and subsequent lines. A phase 2 trial aimed to demonstrate that trilaciclib prevents the hematologic toxicity of chemotherapy, thereby delivering chemotherapy at full doses in time and improving treatment outcomes. The primary endpoint, which was duration of neutropenia, was not met; however, patients in the two groups treated with trilaciclib had longer OS [95]. In the final analysis, the median OS in the groups treated with trilaciclib was 19.8 months compared to 12.6 months in the arm treated with chemotherapy only (HR 0.37, 95% CI 0.2–0.6; *p* < 0.0001). The benefit was consistent in all molecular subtypes of TNBC as well as in PD-L1-negative and PD-L1-positive populations [96]. Following these results, the PRESERVE-2 phase 3 trial was designed and is comparing trilaciclib or placebo in addition to gemcitabine/carboplatin chemotherapy in first- or second-line treatment of mTNBC [97, NCT04799249]. Table 3 summarizes targeted therapy in TNBC.

**Table 3:** Targeted therapy in triple-negative breast cancer

Drug [Ref.]	Target	No. of patients	Study design	Study phase	Results
Olaparib	PARP				
[63]		302 (150 TNBC)	Previously treated metastatic TNBC and HR positive HER2 negative breast cancer, germline BRCA 1/2 mutated olaparib *vs*. investigator choice of therapy (capecitabine, vinorelbine, eribulin)	3	PFS (ITT) 7.0 *vs*. 4.2 months, HR 0.58 95% CI 0.43–0.80; *p* < 0.001. PFS (TNBC) HR 0.43 95% CI 0.29–0.63; OS (ITT)19.3 *vs*. 19.6 months 95% CI 0.63–1.29; *p* = 0.57.
[66,67]		1836 (1509 TNBC)	Adjuvant TNBC or hormone receptor positive, HER2 negative, germline BRCA 1/2 mutated olaparib *vs*. placebo (1 year)	3	3-year IDFS 85.9% *vs*. 77.1%, *p* < 0.001, HR 0.58 99.5% CI 0.41–0.82. 4-year OS 89.8% *vs*. 86.4%, HR 0.68 95% CI 0.47–0.97; *p* = 0.009.
Talazoparib	PARP				
[64]		431 (190 TNBC)	Previously treated metastatic TNBC and HR positive HER2 negative breast cancer, germline BRCA 1/2 mutated talazoparib *vs*. investigator choice of therapy (capecitabine, vinorelbine, eribulin, gemcitabine)	3	PFS 8.6 *vs*. 5.6 months, HR 0.54, 95% CI 0.41–0.71; *p* < 0.001. OS 19.3 *vs*. 19.5 months, HR 0.84, 95% CI 0.670–1.073; *p* = 0.17.
Veliparib	PARP				
[65]		513	Previously treated metastatic TNBC and HR positive HER2 negative breast cancer, germline BRCA 1/2 mutated velaparib+paclitaxel/carboplatin *vs*. placebo+paclitaxel/carboplatin	3	PFS 14.5 *vs*. 12.6 months, HR 0.71 95% CI 0.57–0.88; *p* = 0.0016.
[69]		634	Neoadjuvant TNBC velaparib+carboplatin+paclitaxel *vs.* carboplatin+paclitaxel *vs.* paclitaxel	3	pCR (velaparib+carboplatin+paclitaxel *vs*. paclitaxel) 53% *vs*. 31%, *p* < 0.0001. pCR (velaparib+carboplatin+paclitaxel *vs*. carboplatin/paclitaxel) 53% *vs*. 58%, *p* = 0.36.
Ipatasertib	AKT			
[76]		124	Untreated TNBC ipatasertib+paclitaxel *vs*. placebo+paclitaxel	2	PFS (ITT) 6.2 *vs*. 4.9 months, HR 0.60, 95% CI 0.37–0.98; *p* = 0.037. PFS (PTEN low) 6.2 *vs*. 3.7 months, HR 0.59; 95% CI 0.26–1.32; *p* = 0.18.
[77]		151	Neoadjuvant TNBC ipatasertib+paclitaxel *vs*. placebo+paclitaxel	2	pCR (ITT) 17% *vs*. 13% pCR (PTEN low) 16% *vs*. 13% pCR (PIK3CA/AKT1/PTEN alterated tumors) 18% *vs*. 12%.
[78]		255	Untreated metastatic TNBC ipatasertib+paclitaxel *vs*. placebo+paclitaxel	3	PFS 7.4 *vs*. 6.1 months, HR 1.02 95% CI 0.71–1.45.
Capivasertib	AKT				
[79]		140	Untreated metastatic TNBC capivasertib+paclitaxel *vs*. placebo+paclitaxel	2	PFS (ITT) 5.9 *vs*. 4.2 months, HR 0.74; 95% CI 0.50–1.08; *p* = 0.06. OS (ITT) 19.1 *vs*. 12.6 months, HR 0.63; 95% CI 0.37–0.99; *p* = 0.04, PFS (PIK3CA/AKT1/PTEN alterated tumors) 9.3 *vs*. 3.7 months, HR 0.30; 95% CI 0.11–0.79; *p* = 0.01.
Trilaciclib	CDK4/6				
[90,91]	102		Previously treated TNBC trilaciclib+gemcitabine/carboplatin *vs*. placebo+gemcitabine/carboplatin	2	Duration of severe neutropenia not significantly different OS 19.8 *vs*. 12.6 months, HR 0.37, 95% CI 0.2–0.6; *p* < 0.0001.

Note: TNBC-triple negative breast cancer, atezo-atezolizumab, PFS-progresion free-survival, OS-overall survival, EFS-event free-survival, IDFS-invasive disease free-survival, DDFS-distant disease free-survival, pCR-pathological complete response, pembro-pembrolizumab, durva-durvalumab, gem-gemcitabine, carbo-carboplatin.

### Other druggable targets

NTRK 1–3 fusions encode proteins that are oncogenic drivers in many tumors [98]. In TNBC, NTRK fusions are rare (0.1–0.2%); however, in secretory breast cancers, they are present in over 90% of cases [98,99]. In basket trials, entrectinib and larotrectinib achieved significant ORR in different tumor groups [100,101]. The MAP kinase signaling pathway is altered in approximately 3% of TNBC cases [102]. The TORCMEK study was a phase 1b/2a study that combined the MEK1/2 inhibitor, selumetinib, with the mTORC1/2 inhibitor, vistusertinib, in various solid tumors. The result of this signaling pathway blockade led to stabilization of the disease for more than 16 weeks in 7 out of 23 treated patients. Five patients with TNBC were also included in the study [103].

The SUMMIT study is a phase 2 basket study investigating neratinib in various tumors with HER2 mutations. In TNBC, HER2 mutations are present in approximately 3% of cases [99,104]. The efficacy of olaparib was investigated in the TBCRC 048 phase 2 study, which included 54 patients with metastatic HER2-negative breast cancer and somatic BRCA 1 or 2 mutations or germline pathogenic variants in PALB2, ATM, and CHEK2. Most of the included patients (76%) had HR+/HER2-disease. Cohort 1 included patients with non-BRCA1/2 germline pathogenic variants, while cohort 2 included patients with somatic BRCA and other somatic mutations. The ORR of cohort 1 was 33%, while the ORR in cohort 2 was 31%. Responses were only reported in those patients with germline PALB2 pathogenic variants (ORR: 82%) and somatic BRCA1/2 mutations (ORR: 50%) [105]. Although rare mutations, the above studies support the increasing need for personalized therapy of TNBC and the potential clinical value of applying next generation sequencing in the treatment of breast cancer [106].

**Figure 1:**
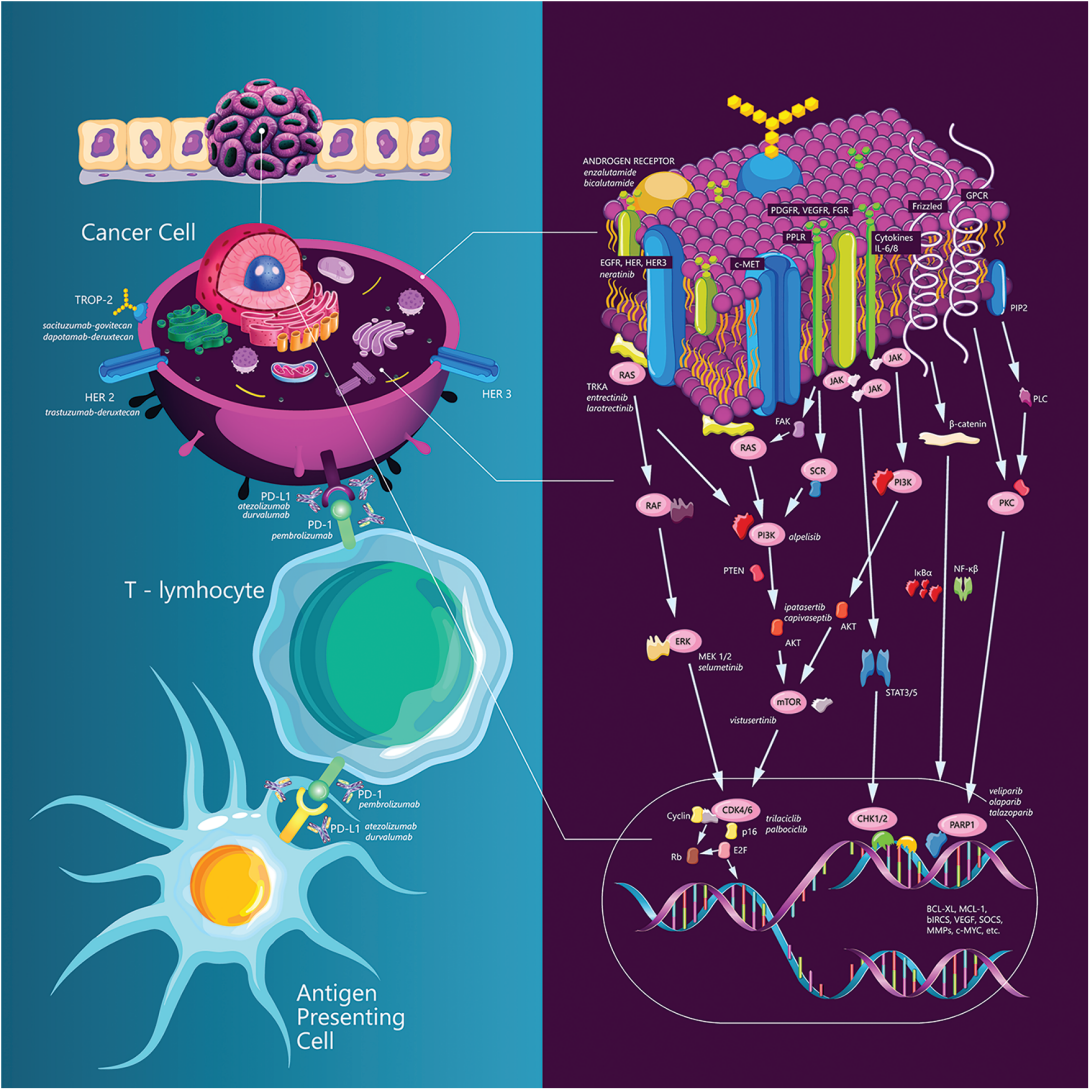
TNBC targeting strategies.

Fig. 1 summarizes TNBC targeting strategies.

## Novel Roles of Chemotherapy

Chemotherapy is still the most commonly used type of therapy in the treatment of both early and metastatic TNBC. Standard neoadjuvant therapy includes anthracyclines and taxanes [107]. A dose-dense schedule, being particularly effective for TNBC, is the preferred treatment option and is associated with OS benefit [108,109].

A recent meta-analysis that included nine studies comparing the addition of carboplatin to standard neoadjuvant therapy indicated a higher pCR rate with carboplatin (52.1% *vs*. 37%). In addition, studies were selected that specifically analyzed the addition of carboplatin in patients with gBRCA1/2 mutations. In this small subgroup of patients, pCR rates were high in both groups (58% *vs*. 56.2% in the platinum group compared to standard therapy), indicating the sensitivity of gBRCA1/2 mutations to chemotherapy in general and not exclusively to platinum compound [110]. More recently, updated survival results have demonstrated a significant improvement in event-free survival (HR 0.70; 95% CI 0.56–0.89) and a trend for OS (HR 0.82; 95% CI 0.64–1.04) [111]. The role of carboplatin in adjuvant treatment has not been extensively studied. The PATTERN study showed a longer DFS in the carboplatin/paclitaxel combination compared to the standard sequential regimen of anthracyclines and taxanes (5-year DFS: 86.5% *vs*. 80.3%; *p* = 0.03, HR 0.65); however, it did not address the objective of platinum addition [112]. The mentioned BrightNess trial compared paclitaxel with the addition of carboplatin and the addition of carboplatin and veliparib as neoadjuvant therapy [74]. The study showed the benefit of the addition of carboplatin in terms of an increased pCR rate. The addition of veliparib was not associated with improved outcomes. In an updated analysis of EFS after 4.5 years of follow-up, the addition of carboplatin to paclitaxel resulted in a significantly longer EFS (HR 0.57, 95% CI 0.36–0.91, *p* = 0.02) without a significant impact on OS. The addition of veliparib did not contribute to a longer EFS [113].

CREATE-X is a study that evaluated the benefit of the addition of capecitabine in the adjuvant setting in HER2-negative cancer with residual disease after neoadjuvant chemotherapy. In the TNBC group, the 5-year DFS was 69.8% and 56.1% in the capecitabine and placebo arm, respectively, and the OS was 78.8% and 70.3% in the capecitabine and placebo arm, respectively [114]. Additionally, the SYSUCC-001 study demonstrated a longer 5-year DFS if low-dose capecitabine for one year was added to standard adjuvant therapy [115].

Recently, published results of the ECOG-ACRIN EA1131 phase 3 trial, evaluating platinum chemotherapy compared to capecitabine in the adjuvant setting in TNBC patients with residual disease after neoadjuvant chemotherapy and surgery, demonstrated that platinum chemotherapy (cisplatin/carboplatin) did not improve invasive DFS (iDFS) in comparison with capecitabine in these patients. The three-year iDFS was 42% and 49% for platinum chemotherapy and capecitabine, respectively, with higher toxicity of platinum compounds [116].

## Treatment Pattern and Open Questions

In early-stage TNBC, neoadjuvant therapy is the standard of care for all patients, except in those with node-negative disease and small tumor size. After the results of the Keynote-522 study, pembrolizumab in combination with anthracyclines, paclitaxel, and carboplatin is the standard treatment in the neoadjuvant-adjuvant setting, except for patients with node-negative disease with a tumor size smaller than 2 cm [18]. Two questions remain open. First, it is unknown whether patients unfit for platinum- or anthracycline-based chemotherapy should receive pembrolizumab, or whether we can extrapolate these results from the addition of pembrolizumab to other chemotherapy backbones (including those administering the anthracycline part in a dose-dense manner). The second complex question is to understand which adjuvant therapy to apply in patients without pCR after pembrolizumab-based neoadjuvant therapy. In BRCA1/2-mutated patients, olaparib demonstrated a benefit in overall survival if pCR was not achieved after neoadjuvant chemotherapy without pembrolizumab [71]. Despite the lack of scientific evidence to support the efficacy of olaparib-pembrolizumab combination but in light of the availability of safety data in the advanced setting and evidence of benefit for both treatments in a clinical setting with high risk for disease relapse, our suggestion would be to combine pembrolizumab and olaparib in BRCA1/2 mutated patients who did not achieve pCR, particularly in patients with residual cancer burden II and III. If this combination is not possible, olaparib should be the preferred option. The same approach would apply for the use of capecitabine in patients who did not achieve pCR and do not harbor BRCA1/2 mutations. Although the results are promising [19,20,33], the evidence is insufficient thus far to recommend the use of atezolizumab and durvalumab in early TNBC.

In the treatment of mTNBC, chemotherapy is the first-line treatment for all patients. In approximately 40% of those with PD-L1-positive tumors, the combination of CPI and chemotherapy results in prolonged survival. In the USA, due to the withdrawal of the label of atezolizumab, the standard treatment is the combination of pembrolizumab with chemotherapy (paclitaxel, nab-paclitaxel, or gemcitabine/carboplatin) in patients with PD-L1 CPS ≥10. In Europe, both atezolizumab in combination with nab-paclitaxel and pembrolizumab in combination with the abovementioned protocols are registered for the treatment of mTNBC. It is important further investigate PD-L1 testing in terms of which tissue to test and which antibody to use. Giugliano et al. [117] suggested that more research is needed to harmonize PD-L1 testing in mTNBC as well as to determine which specific diagnostic antibody with a specific drug should be used. Future studies also warrant to further investigate other protentional predictive factors beyond PD-L1 expression of ICI response in the mTNBC [118]. Another open question is to understand whether immunotherapy should be reused in the treatment of mTNBC if the patient previously received immunotherapy in the early setting. From the second line treatment onward, sacituzumab govitecan is the preferred systemic treatment option whenever available as it prolongs OS compared to chemotherapy. Second and subsequent lines of treatment for TNBC may also be chemotherapy or PARP inhibitors (olaparib or talazoparib) in BRCA1/2-mutated patients as well as T-DXd in HER-low patients. ESMO recommends NGS-based personalized therapy when all standard therapy options have previously been used [50]. Importantly, the utilization of NGS in breast cancer is increasing year by year [106]. Other targeted therapies and androgen receptor blockers still lack sufficient evidence to be recommended in the treatment of TNBC.
